# Placebo in Surgical Research: A Case-Based Ethical Analysis and Practical Consequences

**DOI:** 10.1155/2016/2627181

**Published:** 2016-08-10

**Authors:** Sorin Hostiuc, Irina Rentea, Eduard Drima, Ionut Negoi

**Affiliations:** ^1^Department of Legal Medicine and Bioethics, Faculty of Medicine, Carol Davila University of Medicine and Pharmacy, Bucharest, Romania; ^2^National Institute of Legal Medicine, 042122 Bucharest, Romania; ^3^Faculty of Medicine, Carol Davila University of Medicine and Pharmacy, 020021 Bucharest, Romania; ^4^University of Medicine and Pharmacy, 800216 Galați, Romania; ^5^Galați Psychiatry Hospital, 800216 Galați, Romania; ^6^Department of Surgery, Faculty of Medicine, Carol Davila University of Medicine and Pharmacy, 020021 Bucharest, Romania

## Abstract

Placebo is a form of simulated medical treatment intended to deceive the patient/subject who believes that he/she received an active therapy. In clinical medicine, the use of placebo is allowed in particular circumstances to assure a patient that he is taken care of and that he/she receives an active drug, even if this is not the case. In clinical research placebo is widely used, as it allows a baseline comparison for the active intervention. If the use of placebo is highly regulated in pharmacological trials, surgery studies have a series of particularities that make its use extremely problematic and regarded less favorably. The purpose of this paper is to present three famous cases of placebo use in surgical trials and to perform an ethical analysis of their acceptability using the Declaration of Helsinki as a main regulatory source.

## 1. Introduction

Placebo is a form of simulated medical treatment intended to deceive the patient/subject who believes that he/she received an active therapy. In clinical medicine, the use of placebo is allowed in particular circumstances to assure the patient that he is taken care of and that he/she receives an active drug, even if this is not the case [1]. Placebo is useful for particular pathologies, including depression, anxiety, or surgical related pain [2, 3]. However, some systematic reviews and meta-analyses recently demonstrated that the placebo effect is subjective. For example, Hróbjartsson and Gøtzsche showed that if a study had a binary outcome (patients improved/not improved after administering placebo), or if the end results were objective (like the values of blood pressure), placebo had no statistically significant positive effect. However, when the result was subjective (reported by the subjects), it seemed that placebo had a statistically significant positive effect [4].

The use of placebo is limited in clinical medicine on moral grounds, as it breaches the trust of the patient in his/her physician (the latter has to lie about the chosen therapeutic alternative). For this reason, ethically, placebo use is allowed only in particular circumstances. Siegler, for example, considers that placebo can be used in clinical practice if four conditions are simultaneously met: (1) the condition is known to respond well to placebo, (2) the alternative to placebo is either continued illness or the use of a drug with known adverse risks or addiction, (3) the patient wishes to be treated, and (4) the patient insists on obtaining a prescription from the physician [5].

Experimental clinical medicine is nowadays fundamentally based on randomized clinical trials, considered the most reliable form of scientific evidence in healthcare and an essential element for the development of evidence-based medicine. Randomized clinical trials consist of comparing a new intervention with a control that usually is either the best-alternative-treatment (BAT) for that specific pathology or an inactive intervention (placebo). Most research ethics codes specifically state that if there is a therapeutic alternative, it should be used for the control group, as this minimizes the potential harm done through nonintervention. For example, the Declaration of Helsinki states the following: “Art 32. The benefits, risks, burdens and effectiveness of a new intervention must be tested against those of the best-proven intervention(s), except in the following circumstances: (a) Where no proven intervention exists, the use of placebo, or no intervention, is acceptable; or (b) Where for compelling and scientifically sound methodological reasons the use of any intervention less effective than the best proven one, the use of placebo, or no intervention is necessary to determine the efficacy or safety of an intervention (c) and the patients who receive any intervention less effective than the best-proven one, placebo, or no intervention will not be subject to additional risks of serious or irreversible harm as a result of not receiving the best-proven intervention. Extreme care must be taken to avoid abuse of this option” [6].

Investigators use placebo to differentiate the real effect, caused by the active intervention, from subjective effects, caused by the belief of the subject that he/she receives an intervention. A major advantage of placebo-controlled trials compared to randomized trials, in which the control is BAT, is the difference in effect sizes between the cases and control groups. Compared to BAT, in placebo-controlled trials the effect size is higher, and the number of subjects needed to reach a certain statistical power is lower. Other reasons in favor of placebo-controlled trials include: (1) a new therapy might not be better concerning the primary outcome compared with the best available therapy, but it might be advantageous in other ways (safety, compliance, tolerability, and cost), (2) the best available therapy control might show an inconsistency in effects caused by methodological differences (e.g., the inclusion criteria that might be different compared to the ones used to prove its usefulness), (3) the presence of methodological limitations in using the best available therapy option [7]. For these reasons, researchers tend to prefer placebo instead of the best alternative randomized controlled trials and often try to “bend the rules” and develop research protocols using placebo when an alternative design could be designed.

The use of placebo in surgery trials is even more controversial. Clark, for example, considers that, unlike placebo-controlled trials in pharmacological research, sham surgeries “fail the test of beneficence” [8]. Weijer gives some compelling arguments for this, including the absence of a therapeutic purpose, significant scientific disadvantages, and a significant risk increase [9]. Some authors developed specific ethical frameworks for the use of sham procedures in trials. For example, Horng and Miller provided an ethical framework in six steps for assessing the acceptability of sham surgeries that included the following: “(1) there is a valuable, clinically relevant question to be answered by the research, (2) the placebo control is methodologically necessary to test the study hypothesis, (3) the risk of the placebo control itself has been minimized, (4) the risk of a placebo control does not exceed a threshold of acceptable research risk, (5) the risk of the placebo control is justified by valuable knowledge to be gained, and (6) the misleading involved in the administration of a placebo control is adequately disclosed and authorized during the informed consent process” [10].

Most studies in this area focused their attention on a single sham surgery study, and the ethical analysis was often directed to the particularities of that particular trial. Moreover, most studies analyzed how to modulate the research methodology to develop an ethical sham surgery study and not if such a study, per se, should be even considered ethical [10].

The purpose of this article is to present three famous cases of placebo use in surgical trials and to perform an ethical analysis of their acceptability in accordance with the Declaration of Helsinki.

## 2. Relevant Cases

### 2.1. Cobb and the Treatment of Angina Pectoris

At the end of the 1950s, the most popular treatment for angina pectoris was the bilateral ligation of the internal mammary arteries [11, 12]. For Doctor Cobb and his colleagues, the results of the treatment were doubtful, and they decided to submit the surgery to a double-blind controlled trial. Seventeen patients were selected to participate in the study. All of them had debilitating symptoms caused by angina, many of them being unemployed. Their physical performance was evaluated before surgery. Also, the investigators performed tests for “respiratory efficiency”, blood pressure, and an electrocardiogram (at rest, during physical activity, and recovery). The physicians told the patients that the ligation was not shown to be effective, but they were optimistic. They were not informed of being included in a controlled trial nor that some of them might not receive the actual procedure. The sham surgery consisted of local anesthesia and a skin incision [13]. Of the seventeen patients, eight underwent the initial procedure and nine the sham surgery. The patients were evaluated after the surgery in a period of three to fifteen months. Five out of 8 patients who underwent the full surgery and 5 out of 9 patients who underwent the sham surgery had “significant” subjective improvement. Only two patients had an actual improvement when comparing the results of the exercise tests before and after the operation. These two subjects underwent the sham surgery. The conclusion of the study was that there was no significant improvement in the patients who had the full surgery compared to the sham procedure.

### 2.2. Moseley and the Arthroscopic Surgery for Osteoarthritis of the Knee

To the writers of the 2002 study, “A Controlled Trial of Arthroscopic Surgery for Osteoarthritis of the Knee”, the physiological basis for the pain relief of the surgery was unclear [14]. The lack of evidence that the surgery cured or slowed down osteoarthritis led them to design a double-blind controlled trial aimed at assessing its efficiency. One of the reasons behind this study was that 650.000 procedures took place every year at an approximate cost of $5000 each [15]. The study was approved by relevant Institutional Review Board (IRB), and the data regarding the safety of the procedures was monitored throughout the trial. The investigators found 324 patients that fulfilled the inclusion criteria (75 years old or less, a diagnosis of osteoarthritis of the knee, according to the criteria at the time [16], moderate pain in the knee, and not having had this surgery in the last two years). 56% of the eligible patients agreed to participate in the study [14]. The patients were divided into three groups by the severity of their osteoarthritis through a stratified randomization process. The subjects were randomly assigned to one of the following procedures: arthroscopic debridement, arthroscopic lavage only, or the placebo procedure. They were informed that they may only receive the placebo and that, if included in this cohort, they would have no benefit from the surgery. The surgeons who did the intervention were not involved in the follow-up procedure to keep the double-blind nature of the study. The sham surgery consisted of making three skin incisions. When the patients were later asked to guess if they had the actual operation or the placebo, 86.2% of the patients from the sham surgery cohort thought that they had the real procedure, and 86.8% of the patients who received the actual operation guessed correctly. The study concluded that arthroscopic surgery had no higher positive effect compared to sham surgery and that the funds used for the procedure could have a better destination. It also showed that placebo surgery actually could have a great positive impact on the patient's health even if its mechanism of action is unclear. One important thing to note is that even though this study came out in 2002, 14 years before the writing of this paper, the procedures described in the study are still in use [17, 18].

### 2.3. Use of Fetal Stem Cells for Parkinson's Disease

In the early 2000s in the medical community appeared a new idea regarding the treatment of Parkinson's disease (PD), namely, the transplantation of fetal tissue in the brain of the patients. Open clinical trials showed that patients experienced some benefits from the procedure, but their magnitude was not clear. Some researchers from the University of Colorado School of Medicine decided to run a double-blind placebo-controlled trial to determine if the benefit was greater than placebo. They selected 40 patients, 34- to 75-year-olds with severe Parkinson's disease to participate in the trial. To preserve the impression that the operation took place for the placebo group, the investigators drilled holes in the skulls of the subjects, without penetrating the dura. The patients were followed for a year after surgery. The physicians who did the follow-up did not know if their patients had the sham or the actual surgery. The consent form detailed the risks and potential benefits, and it was approved by the IRB at the University of Colorado, Columbia University, and North Shore University Hospital. It is not clear if the patients were aware that they could receive sham surgery. The investigators obtained fetal tissue after getting a written consent from the women who requested the abortion procedure. In patients who had the surgery and were younger than 60, there was a significant improvement as measured by standardized Parkinson's tests. However, five patients with transplant showed late dystonia and dyskinesia, suggesting the procedure still needed refinement [19].

## 3. Discussions

To assess the ethical acceptability of the sham surgeries, we will analyze some arguments for and against this procedure and will try to evaluate their validity critically within the ethical framework established by the Declaration of Helsinki.

These three cases have been selected as many authors consider them the most representative for sham surgery and its ethical implications. Each led to a plethora of articles or books discussing the ethical ramification of the studies [20–37]. Most studies analyzed a single case, a fact that could lead to a biased analysis of this issue, based on the particularities of that sham surgery study. Miller performed an ethical analysis of the same three studies presented here [38], which was founded on the six key ethical questions established by Horng and Miller [10]. Their analysis does not fully take into account the principles stipulated in the Helsinki Declaration, nor the EU norms, making it of a limited usefulness outside the US. The first two general principles of the Helsinki Declaration clearly state the following: “Art 3. The Declaration of Geneva of the WMA binds the physician with the words, ‘The health of my patient will be my first consideration,' and the International Code of Medical Ethics declares that ‘A physician shall act in the patient's best interest when providing medical care.' Art 4. It is the duty of the physician to promote and safeguard the health, well-being, and rights of patients, including those who are involved in medical research. The physician's knowledge and conscience are dedicated to the fulfillment of this duty” [39]. We believe that these principles should be the basis of the analysis regarding the ethical acceptability of any clinical trial. Within the patient-subject duality, the patient should be always seen as the most important part and subsequently, within the physician-investigators duality, this role should be taken by the physician. This principle ensures the continuous trust of the patient in his physician in particular and in healthcare in general. Without it, patients would not trust that, when recommending their inclusion in a clinical trial, physicians truly have their best interest at heart. Therefore, we think that the first step in any potential placebo-controlled surgical trial should be the assessment of the acceptability of that particular type of study by the possible subjects. This evaluation should be unbiased and should truly reflect the opinions of the patients.

### 3.1. Is Sham Surgery Accepted by Potential Subjects?

Frank et al. performed a study on subjects with and without Parkinson's disease to assess their willingness to participate in neurosurgical trials for this disease. The investigators selected three groups of patients: with PD, without PD but with other neurological diseases (dementia excluded), and patients from primary care. They then gave each the option to select from the following: to be included in an unblinded trial, to be included in a blinded trial, or not to participate. Most subjects from each group selected to participate in the unblinded trial (range from around 55% in non-PD patients to 41.5% in PD patients). The highest number of subjects selecting nonparticipation was in the PD group (34%, while in the other two the maximum nonparticipation portion accounted for 10.4%). Also, the PD group of subjects was the least willing one to be involved in a blinded study (24.5%, while the other groups favored this option in a percent from 35 to 40%) [24]. The authors concluded that “patients with PD, when compared with patients with non-PD neurology or primary care, may have adapted to their chronic illness and may not be so desperate that they would be more eager to participate in risky research. In fact, they appear more cautious” [24]. This conclusion is, of course, subjective and not based on the actual study. Maybe, for example, the PD patients did not want to be included in the blinded trial because they were directly affected by the procedure and felt the risks were too high. Whenever we would like to analyze the opinions of patients regarding a certain medical procedure, we must take into account all the possible reasons for a certain response, and that their replies are in line with their actual beliefs. Such patient surveys tend to be more and more performed and used as objective proof, suggesting that patients agree with more controversial issues, not taking into account the validity of the used questionnaires or the mere fact that, for a certain procedure to be decided, the individual consent and not a population agreement regarding its usefulness is needed. Moore et al., in the TransEuro project, found that subjects enrolled in PD clinical trials tend to be more educated, younger, with a higher cognitive score, and better motor function compared to patients that were eligible, but not included in the trial, and argued that this could raise problems regarding a parity of access to clinical trials [40]. However, this could also be a method of protecting vulnerable subjects. According to the Helsinki Declaration, “Medical research with a vulnerable group is only justified if the research is responsive to the health needs or priorities of this group and the research cannot be carried out in a non-vulnerable group. In addition, this group should stand to benefit from the knowledge, practices or interventions that result from the research” [39]. Therefore, this study complies with the provisions regarding the protection of vulnerable populations from the Helsinki Declaration. Swift, in a qualitative study about the perspective of the patients and their relatives about sham surgery in PD, showed that participation was acceptable for a small majority of interviewers, but the main reasons for accepting it seems to be the severity of the disease and the lack of good treatment options. Moreover, the surveyed persons preferred real to sham surgery; this comes to support the idea that subjects see themselves primarily as patients, that acceptance for participating in clinical trials is not based on altruistic reasons, and that therapeutic misconception might be significant [21].

### 3.2. Sham Surgery as a Form of Mitigated Trolley

Albin considers that sham surgery can be regarded as a form of mitigated trolleys. The trolley problem (and other similar examples) is often used to test the moral intuition for circumstances in which a few people are put at risk to save more. In this problem, a runaway trolley goes down a track towards five men who will be killed if it is not stopped or diverted. The trolley cannot be stopped, but it can be diverted. However, on the secondary line, there is another person who will be killed by the trolley. So save five and kill one by acting or save one and kill five by not acting? Albin considers that sham surgery can be partially assimilated with a trolley in which the conductor diverts the line but puts a padding on the front to cushion the impact of the trolley. The decision similar to the one made by the trolley conductor is to perform a clinical trial, and subsequently to put some people at a mitigated risk (the surgery is partially simulated, so cushioned) to aid many [41]. What is wrong with this approach? Even if it apparently leads to a maximization of the benefits and is often used by the supporters of utilitarian ethics in healthcare, we believe that it contradicts the utilitarian moral theory. Bentham and Bowring, in their book *Deontology of the Science of Morality*, said that an action is correct or incorrect, deserving or not, receiving approval or disapproval, reported to the tendency in which it causes the increase or decrease in the quantity of public happiness [42]. This means that when we analyze whether an act is moral or immoral from a utilitarian perspective, we should examine not only the good done to the ones directly affected by our actions but also the one generated by them in the general population. If a physician saves a few lives at the expense of sacrificing one, he does apparently more good directly; however, his actions might cause a decrease in the trust in physicians in general—why should I, as a patient, go to a doctor if he might sacrifice me for the good of others? This decrease in trust would cause a decreased addressability of patients toward the healthcare system and a decreased therapeutic compliance, therefore causing more harm overall.

### 3.3. The Risk-to-Benefit Analysis

One of the major reasons for accepting sham surgeries is represented by the fact that minor risks for a few patients are considered to be less important than the overall potential benefit the results of the study might lead to, for both the subject and the population potentially benefit from it. The potential benefit for Parkinson's patients is potentially significant if the therapy will actually have a positive clinical effect. The question is how would the subjects from the control group benefit from it. Normally, if the procedure is shown to be useful, they would receive the same procedure after the end of the trial; therefore, apparently, they would benefit from all the positive results of the trial without risking any unforeseen complication generated by the implantation of fetal stem cells in their brains. However, for this purpose, they would suffer two surgical interventions (one for the trial and one for the therapy), which might be associated with significant risks, especially taking into account the fact that most patients are old, with a severe pathology and subsequently have a higher surgical risk. In the arthroscopy trial, the potential benefit was less certain—in theory, the subjects with arthritis pain might not need another surgical intervention. However the risks were minor compared to the Parkinson's trial; the subjects were mostly younger, the surgical intervention was less invasive (only cutaneous incisions compared to drilling holes in the skull), and the anesthetic risk was in general lower. So apparently we have two studies in which the risk-to-benefit ratio is quite similar. In instances such as this, we should take primarily into consideration the potential risks for the patients (first do no harm, as stated in the Hippocratic works). The risks in the Parkinson's trial are larger compared to the arthroscopy study; in the arthroscopy trial, they are minor. Therefore, we think that from this point of view the arthroscopy trial was ethically permissible, while the Parkinson's was not. Weijer even considers that sham surgical procedures should be analyzed as nontherapeutic interventions and argues that a nonsurgical control is likely a better option. According to him, therapeutic procedures should pass a test of clinical equipoise, and for them should be performed a harm-to-benefit analysis. Nontherapeutic procedures do not offer the prospect of benefits to the individual, and therefore, the harm-to-benefit analysis is not appropriate. For them this analysis should be replaced with two others, the minimization of the risks consistent with sound design and the reasonability of the risks in relation to the knowledge to be gained [9]. By using the above two mentioned principles regarding risk analysis, which is in strict accordance with the Declaration of Helsinki (Art. 17), the use of sham surgery should be forbidden. Minimization of risks, in nontherapeutic interventions, cannot be correlated with the magnitude of the benefit; therefore, any risks that are more than minimal are in contradiction with the principle of nonmaleficence as established by the Declaration of Helsinki.

### 3.4. What Role Should Have Collateral Benefits in the Decision to Allow Sham Surgery?

Besides a direct benefit for the subjects, derived from the actual therapeutic intervention, in clinical trials the possibility for them to receive collateral benefits is sometimes discussed, generated by the inclusion in the trials—a case in which all subjects benefit from the best possible treatment [43]. King considers that collateral benefits should not be used as types of benefits included in the risk analysis of clinical trials for two main reasons: (1) by providing a potentially higher standard of care for subjects compared to patients, we might potentially discourage the improvement of the standard treatment, and (2) as collateral benefits are under the control of the investigators/sponsors, they might become a means of manipulating or even coercing vulnerable patients to enter the clinical trial [43]. We agree with her opinion, but we think that these collateral benefits should not be used in the risk-benefit analysis. However, if the study generates such a benefit, the participants should benefit from it, as a reward for their altruistic participation in the study (see also the Declaration of Helsinki, Art 34). Moreover, there are specific means to protect individuals from coercion or manipulation that can be easily imposed by the IRBs, especially related to the pattern of information given to the potential participants.

### 3.5. Are Placebo-Controlled Trials Actually Needed in Surgery Studies?

We presented in Introduction a few reasons for which investigators tend to prefer placebo versus BAT randomized clinical trials. These reasons might cause a slight bend of the strict ethical rules governing the use of placebo in clinical trials by the investigators, in instances in which the BAT could be circumvented. Investigators tend to search ways to circumvent BAT in favor of placebo instead of reconciling the need for statistical power and significance with BATs, in instances in which alternative designs could be developed. For example, Dekkers and Boer argued that for Parkinson's trials an alternate design could consist of a core assessment protocol, in which measurement protocols are applied to the subjects before and after the surgical intervention [34]. Avins argues that unbalanced randomization might be less morally problematic in some instances, with the risk, however, of losing statistical power [44]. Macklin suggested that “cellular-based surgical therapies have much in common with pharmacologic treatments and lend themselves to evaluation in randomized, double-blind, placebo-controlled trial” [35]. Even if from a research methodology point of view this may be true, this is not the case from a bioethics perspective. The risks associated with anesthesia and a surgical procedure are inherently higher than in most pharmacological clinical trials, especially for older individuals with severe associated pathologies. If they are to be accepted by the potential subjects, this is because of an inherent wish to get better and to receive an experimental treatment with significant benefits from a healthcare point of view [9, 21]. Between these two types of therapies are also substantial differences regarding the way concepts like autonomy, therapeutic misconception, or trust, are perceived by the patients. Therefore, we should not try to minimize but rather emphasize the differences between surgical (even is minimally invasive) and clinical trials to reveal their particularities. Only by doing this we could minimize the ethical issues raised in practice by sham surgery.

### 3.6. Autonomy versus Therapeutic Misconception

According to Lidz and Applebaum, therapeutic misconception occurs “when a research subject fails to appreciate the distinction between the imperatives of clinical research and of ordinary treatment, and therefore inaccurately attributes therapeutic intent to research procedure” [45]. The therapeutic misconception may be caused by the patient's expectations that the physician will act in his/her best interest even during a clinical trial by the lack of understanding regarding the concept of randomization, by treatment constraints associated with clinical trials, or by their wish that the study will be beneficent to them [46]. There are two main responses to therapeutic misconception: to accept it as an inevitable consequence of clinical trials or to implement measures whose purpose is to reduce it, including the use of the “neutral discloser,” rewriting of the informed consent forms, changes in the information algorithm used by physicians when trying to enroll a patient in a clinical trial, and changes in monetary rewards or research advertisements [47]. Therapeutic misconception is especially high in fields in which the patients are highly vulnerable like oncology or psychiatry. We believe that surgery should also fit in this category, as a patient programmed for a surgical procedure most likely expects a direct benefit resulting from it. Moreover, sometimes even a proper information might not change his/her preconception regarding the clinical utility of the surgery. Therefore, to minimize therapeutic misconception subjects must be explicitly informed about the sham surgery, unlike in the Cobb's trial, and the acknowledgment by the subjects of this issue should be tested explicitly before they sign the informed consent.

The above-presented list is by no means exhaustive; it shows, however, the complexity of the problem and the difficulties of its ethical analysis. For clinical practice, a series of guidelines have been developed regarding the possibility of using placebo (sham surgeries) in research, of which one of the easiest and most useful for surgical investigators is the one developed by Tenery et al., who based their ethical analysis on the following elements: (a) placebo-controlled trials should only be used in surgery if there are no other designs that could lead to the necessary information; (b) a special care should be given to the obtaining of the informed consent. The potential subjects should clearly know the risk and particularities of each arm of the study, with an emphasis on the interventions that would/would not be performed. It is recommended for a third party (not the investigator) to obtain the informed consent; (c) placebo controls should not be used when the investigators investigates the usefulness of a slightly modified surgical procedure; (d) placebo controls should be allowed when it is developed for a surgical procedure for an affliction for which there is no surgical treatment, or if the efficiency of the standard surgical procedure is questionable, and if it is known that the affliction is potentially influenced by placebo, or if the risks of the placebo intervention are small; (e) if the surgical treatment has high risks, and the standard, nonmedical treatment is efficient and acceptable to the patients, it should be offered in all the arms of the study [48]. Additionally, we believe that a first step should consist of a proper analysis of the acceptability of the sham surgery by potential subjects. Moreover, specific measures should be taken to minimize issues like coercion generated by potential collateral benefits, or therapeutic misconception.

The approach presented in this paper is based mainly on the recommendations regarding clinical research as presented in the Helsinki Declaration. According to it, the patient should always come first, and the physician-patient relationship should always take precedence over the investigator-subject relationship. The medical good of a single patient should be more important for a physician than the good of the community as a whole, and the conflict between his duty to the patient and the one to the community should always be solved in the favor of the patient.
